# The Precautionary Principle in the Context of Mobile Phone and Base Station Radiofrequency Exposures

**DOI:** 10.1289/ehp.0900727

**Published:** 2009-05-18

**Authors:** Mike Dolan, Jack Rowley

**Affiliations:** 1 Mobile Operators Association, London, UK; 2 GSM Association, London, United Kingdom

**Keywords:** electromagnetic fields, precautionary approach, precautionary principle, public concern, scientific uncertainty, technology

## Abstract

**Background:**

No health hazard has been established from exposure to radiofrequency fields up to the levels recommended by the International Commission on Non-Ionizing Radiation Protection. However, in response to public concern and the perceived level of scientific uncertainty, there are continuing calls for the application of the precautionary principle to radiofrequency exposures from mobile phones and base stations.

**Objective:**

We examined the international evolution of calls for precautionary measures in relation to mobile phones and base stations, with particular focus on Australia and the United Kingdom.

**Results:**

The precautionary principle is difficult to define, and there is no widespread agreement as to how it should be implemented. However, there is a strong argument that precautionary measures should not be implemented in the absence of reliable scientific data and logical reasoning pointing to a possible health hazard. There is also experimental evidence that precautionary advice may increase public concern.

**Conclusion:**

We argue that conservative exposure standards, technical features that minimize unnecessary exposures, ongoing research, regular review of standards, and availability of consumer information make mobile communications inherently precautionary. Commonsense measures can be adopted by individuals, governments, and industry to address public concern while ensuring that mobile networks are developed for the benefit of society.

Many expert reviews have concluded that no health hazard has been established from exposure to radiofrequency (RF) fields up to the levels recommended by the International Commission on Non-Ionizing Radiation Protection (ICNIRP 1998), although further research is recommended (International Commission of Non-Ionizing Radiation Protection 2009; Scientific Committee on Emerging and Newly Identified Health Risks 2009; Sienkiewicz and Kowalczuk 2005). As part of the continuing debate over possible unidentified health effects due to RF exposure from mobile phones and their base stations, there has been discussion about the application of the precautionary principle (PP) (Vecchia 2007). Calls for precaution are easy to make, but we argue that in the absence of convincing evidence of a possible health risk, it is far more difficult to justify the application of the PP as part of an evidence-based public policy approach.

## The Precautionary Principle

We do not review the origins of the PP because this topic has been covered thoroughly by others (Daemen 2003; Stokes 2008). The PP has been analyzed as having three components: a harm condition, a knowledge/uncertainty condition, and a remedy (Manson 2002). The United Nations Educational, Scientific and Cultural Organization (UNESCO) along with the World Commission on the Ethics of Scientific Knowledge and Technology (COMEST) brought together an expert group on the PP that developed the working definition shown in Appendix 1. This definition attempts to provide some clarity regarding the elements of harm, uncertainty, and proportionate responses.

COMEST (2005) concludes that “the grounds for concern that can trigger the PP need to be plausible or tenable” and that the scientific uncertainty should be “considerable.” COMEST also suggests that “principle” refers to the philosophical basis of the precaution and that “approach” is its practical application, although in most cases the terms will be closely related. The COMEST report also states what the PP is not:

The PP is not based on “zero risks” but aims to achieve lower or more acceptable risks or hazards. It is not based on anxiety or emotion, but is a rational decision rule, based in ethics, that aims to use the best of the “systems sciences” of complex processes to make wiser decisions. Finally, like any other principle, the PP in itself is not a decision algorithm and thus cannot guarantee consistency between cases. (COMEST 2005, p. 16)

Despite its genesis in environmental law and policy, European institutions have accepted that the PP is not limited to damage to the environment and can be applied to public health issues (Daemen 2003; Stokes 2008). European courts are tending toward a position where, “instead of simply providing by default a justification for action in the face of scientific uncertainty, the precautionary principle is beginning to be understood as a positive obligation to conduct risk assessment and establish proof of harm prior to taking protective measures” (Stokes 2008).

## Application of the Precautionary Principle

It has been argued that risk management when done well should be inherently precautionary through use of effective risk assessment to predict, anticipate, and prevent harm, rather than merely reacting. This requires an element of judgment especially regarding balancing the risk of false positives and false negatives when data are uncertain or the topic is controversial (Hrudey and Leiss 2003).

In 1992, the PP was incorporated into the Treaty on European Union (known as the Maastricht Treaty), by name but without definition (Treaty on European Union 1992). Eight years later, in 2000, the European Commission issued a communication on when and how the PP should be applied, intending this to build a common understanding (Commission of the European Communities 2000). In its communication, the European Commission (Commission of the European Communities 2000; italics in the original) discussed six criteria that must be met for measures where action is deemed necessary:

- *Proportional* to the chosen level of protection,
- *Nondiscriminatory* in their application,
- *Consistent* with similar measures already taken,
- *Based on an examination of the potential benefits and costs* of action or lack of action (including, where appropriate and feasible, an economic cost/benefit analysis),
- *Subject to review*, in the light of new scientific data, and
- Capable of assigning responsibility for producing the scientific evidence necessary for a more comprehensive risk assessment.

These criteria emphasize the need for policies to be evidence based, proportional to the risks to be controlled, and mindful of the costs and benefits of measures. In the European Commission criteria, we do not see a conflict between the PP and scientific risk assessment. The PP provides a basis in risk management for political decisions about the appropriate actions that society determines are necessary once an uncertain but scientifically plausible risk is identified.

## RF Exposure from Mobile Phones and Base Stations

Mobile phones are low-powered RF devices transmitting maximum peak powers in the range of 0.6–2 W (ICNIRP 2008). In use, power control algorithms automatically adjust the transmission power to maintain call quality, maximize system capacity, and extend battery life. The effect of power control is to significantly reduce user exposure during normal use; for example, a 3G device typically transmits at 0.2% of the maximum (ICNIRP 2008). These levels are similar to those of cordless telephones, wireless computer devices, and baby monitors (Valberg et al. 2007). Compliance testing is done at maximum transmit power and has been designed to be conservative for all persons (Christ et al. 2005).

Base stations have transmit power levels from a few watts to = 100 W, depending on the size of the “cell” that they are serving. The exposure from base stations in publicly accessible areas is in the range of 0.002–2% of the ICNIRP guidelines, similar to long-established broadcast radio and television services (Valberg et al. 2007). RF exposure generally decreases with increasing distance from the antenna; however, the exposure at the same ground-level distance from different base stations may differ by four orders of magnitude because of base station parameters and environmental scattering (Neubauer et al. 2007). Therefore, distance is not a reliable surrogate for RF exposure in population health studies or government policies.

## RF Fields and Health: State of the Science

Human RF exposure guidelines developed by the ICNIRP are based on established health effects, are kept under review, and incorporate large margins of safety so that they protect all persons against all known health hazards (Vecchia 2007). The ICNIRP guidelines already contain cautionary elements through their use of reduction factors, assumptions of conditions for maximum transfer of RF energy, and separate consideration of workers and the public (ICNIRP 1998).

Existing RF safety recommendations are fundamentally based on limiting temperature rise–related adverse biological responses due to time-averaged absorbed energy. Some have argued that another measure related to the modulation of the RF signal should be used to set a more restrictive standard—for example, the *BioInitiative Report* (BioInitiative Working Group 2007). There is presently no plausible scientific evidence to adopt another metric (International Commission of Non-Ionizing Radiation Protection 2009; Valberg et al. 2007). Therefore, there is no scientific basis to determine what aspect of RF exposure should be modified, a further challenge to defining precautionary measures. The Electromagnetic Fields Committee of the Health Council of the Netherlands (2008) reviewed the *BioInitiative Report* and concluded:

In view of the way the BioInitiative report was compiled, the selective use of scientific data and the other shortcomings mentioned above, the Committee concludes that the BioInitiative report is not an objective and balanced reflection of the current state of scientific knowledge. Therefore, the report does not provide any grounds for revising the current views as to the risks of exposure to electromagnetic fields. The BioInitiative report argues that any effect of electromagnetic fields on biological systems should be avoided, thereby ignoring the distinction between effect and damage.

Similar conclusions have been reached by EMF-NET (2007) and the Australian Centre for Radiofrequency Bioeffects Research (ACRBR 2008).

## The Perils of Precaution: The Stewart Reports

The United Kingdom has been in the vanguard of countries formally incorporating policy recommendations on mobile phone and base station RF exposures with explicit reference to the PP. The Independent Expert Group on Mobile Phones (IEGMP) was established by the U.K. government in April 1999 “to consider present concerns about the possible health effects from the use of mobile phones, base stations and transmitters, to conduct a rigorous assessment of existing research and to give advice based on the present state of knowledge [and] to make recommendations on further work that should be carried out to improve the basis for sound advice” (IEGMP 2000).

The IEGMP conducted a wide-ranging inquiry including consultation with the public and on 11 May 2000 issued the “Stewart Report” (after the surname of its chairman), which concluded that “the balance of evidence to date suggests that exposures to RF radiation below NRPB [National Radiological Protection Board] and ICNIRP guidelines do not cause adverse health effects to the general population.” The IEGMP report continued:

There is now scientific evidence, however, which suggests that there may be biological effects occurring at exposures below these guidelines. We conclude therefore that it is not possible at present to say that exposure to RF radiation, even at levels below national guidelines, is totally without potential adverse health effects, and that the gaps in knowledge are sufficient to justify a precautionary approach. (IEGMP 2000)

The IEGMP report has been criticized for recommending a precautionary approach while concluding that there was no convincing evidence of harm (Burgess 2002):

The report is contradictory. On one hand it is a model scientific distillation of the state of current knowledge about mobile-phone EMF [electromagnetic fields]. But much of the rest reads like a different report, with its endorsement of the “legitimacy” of public fears about mobile towers.

Here we emphasize that the PP does not aim for “zero risk” (COMEST 2005) and that there are many aspects of human activity that are not “totally without adverse health effects”—for example, transport (including aviation) and hot showers—yet society has consistently carried out risk assessments and adopted levels of acceptable risk.

In January 2005, the NRPB (which became part of the U.K. Health Protection Agency on 1 April 2005) reviewed progress on the IEGMP recommendations and concluded:

Within the UK, there is a lack of hard information showing that the mobile phone systems in use are damaging to health. It is important to emphasise this crucial point. . . . The Board believes that the main conclusions reached in the (IEGMP) Report in 2000 still apply today and that a precautionary approach to the use of mobile phone technologies should continue to be adopted. (NRPB 2004)

Again, there is this seeming contradiction with emphasis on the absence of evidence of possible health risks but a restatement of support for precautionary measures. Despite ongoing scientific reassurance from independent scientific review bodies in the United Kingdom and elsewhere, the reality remains that political experience with public health issues in the United Kingdom such as bovine spongiform encephalopathy (BSE) has caused scientific bodies to be cautious. As Sir William Stewart said in evidence to a House of Commons select committee: “The BSE inquiry impacted on us. Never again will any committee say there is no risk” (para. 25; House of Commons 2001).

## The PP, Cautionary Approaches, and Mobile Communications

The possibility of some unknown long-term adverse health effects (including possible effects on children) has been left open by the reviews and is the subject of ongoing research. However, the primary conclusion that, on the basis of available scientific evidence, the ICNIRP recommendations are protective of public health is robust. Therefore, we argue strongly that application of the PP to mobile communications is not justified because the threshold of scientific plausibility (the COMEST term) has not been crossed and there is no convincing theoretical basis that a hazard is likely to be established in the future (Valberg et al. 2007).

Some agencies have concluded that, on the basis of existing uncertainty, it is reasonable to introduce precautionary measures. These measures may include actions by individuals, governments, and industry. In this respect, the present World Health Organization (WHO 2000) advice for individuals states:

Present scientific information does not indicate the need for any special precautions for use of mobile phones. If individuals are concerned, they might choose to limit their own or their children’s RF exposure by limiting the length of calls, or using “hands-free” devices to keep mobile phones away from the head and body.

The corresponding advice to governments is to adopt science-based guidelines and not to undermine confidence by incorporating additional arbitrary safety factors. The WHO goes on to say that if precautionary measures are to be adopted, they should be introduced as a separate policy that encourages, through voluntary means, the reduction of RF fields by equipment manufacturers and the public (WHO 2000).

## Impacts of EMF Precautionary Communication

It has been argued in the context of the power-frequency EMF health debate that undertaking research and addressing public concern by providing information and public education also constitutes acting in a cautionary manner (Sahl and Dolan 1996). However, on its own, scientific research does not adequately address public concern. This is partly due to the fact that research may take some years to complete, and often there are political pressures to implement more immediate public policy actions. Nevertheless, it is vital that the outcomes from research programs are communicated in a timely, transparent, and understandable manner. The failure of research programs to alleviate public concern is also partly attributable to an increasing distrust of science itself, especially in the United Kingdom (Burgess 2002).

Meaningful and effective risk communication should be a significant part of addressing public concern about RF signals (WHO 2002). The mobile phone industry has been pro-active in implementing measures of this nature, including providing compliance information for mobile phones (Mobile Manufacturers Forum 2008). In addition, voluntary codes of best practice for base station siting have been introduced by mobile network operators in several countries to enable early community and local government consultation in base station location decisions (Australian Communications Industry Forum 2004; Mobile Operators Association 2001). Such approaches improve perceptions regarding transparency but do not always lead to greater acceptance of the base station siting process (Wiedemann and Schütz 2008). National approaches must be tailored to the level of concern and existing administrative traditions.

There is research showing that undertaking precautionary measures for the purpose of reassuring the public sends out mixed messages and actually increases community concern (Barnett et al. 2007; Wiedemann and Schutz 2005). Barnett et al. (2007) examined public understanding of two widely available leaflets produced by the U.K. Department of Health in 2000 after publication of the IEGMP report and found that “precautionary advice was generally interpreted as causing concern rather than providing reassurance. This suggests the need for care around the provision of precautionary advice as part of public health information. It seems clear that providing such advice as a response to public concern is unlikely to reassure.”

Barnett et al. (2007) point out that government health advice implicitly relies on increasing concern if it is intended to change an aspect of people’s behavior. There is a logical fallacy in issuing precautionary advice with the stated aim of decreasing public concern; the consequences of these conflicting policy objectives are well documented for the United Kingdom (Burgess 2002). These findings are consistent with our practical experience that the public understanding of precaution is the need to adopt measures to avoid a real (perhaps low probability) potential harm.

Some stakeholders have suggested that young children’s use of mobile telephony should be reduced as a precautionary measure (e.g., IEGMP 2000), because harm might be established in the future. It seems to us that such judgment should be made by parents on a personal basis for their own children and that communication of recommendations should take account of the above findings on perception of precautionary advice. Also, anxiety itself can have deleterious health consequences (Petrie et al. 2001).

## Judicial Approaches to Precaution in Base Station Siting

The courts have taken a conservative approach to precaution in the area of base station siting, giving prominence to the importance of science-based RF exposure guidelines. For example, in Australia the legal position on the application of the PP to base station siting was thoroughly addressed by the chief judge of the New South Wales Land and Environment Court (Telstra Corporation Limited *v.* Hornsby Shire Council 2006). Justice Preston concluded that the PP should not be used to try to avoid all risks (paragraph numbers are from the judgment):

138 If there is not a threat of serious or irreversible environmental damage, there is no basis upon which the precautionary principle can operate. The precautionary principle does not apply, and precautionary measures cannot be taken, to regulate a threat of negligible environmental damage.

He went on to outline the many inherently precautionary elements of the base station proposal:

188 In the present case, such a precautionary approach has already been undertaken, first, in the standard-setting process . . . secondly, in the adoption of the Australian Standard RPS3 [Radiation Protection Series 3] with margins of safety, thirdly, in the requirements of the relevant industry code to comply with the adopted standard, fourthly, in the measurement of existing and the estimation of predicted RF RPS3 levels from the proposed base station . . . fifthly, in the selection of equipment and antennas to be used in the proposed base station and, finally, in the efficient operation of the equipment and antennas to minimise RF EME [electromagnetic energy] levels generated from the proposed base station.

The court declined to accept unfounded community fears as a basis for refusing the development application.

208 Responsiveness to public fear should be complemented by a commitment to deliberation in the form of reflection and reason giving. If the public is fearful about a trivial risk, a deliberative democracy should not respond by reducing that risk. Rather, it should use its institutions to dispel public fear that is, by hypothesis, without foundation. In this way, deliberative democracies avoid the tendency of popularist systems to fall prey to public fear when it is baseless. They use institutional safeguards to check public panics.

This last paragraph should be a reminder to regulators in all countries of the importance of rational and science-based approaches to respond to public fears about radio signals (Rowley 2008). In other legal cases relating to mobile phone base station siting in Australia, New Zealand, and the United Kingdom, the courts have decided that in the absence of credible evidence of risk, compliance with existing exposure guidelines is an appropriate cautionary approach (Optus *v.* CC Kensington and Norwood & Frost, 1998; Shirley Primary School *v.* Telecom Mobile 1998; T Mobile and Others *v.* First Secretary of State and Harrogate Borough Council 2004). This approach is consistent with the general principles enunciated by the European courts and the European Commission (Commission of the European Communities 2000; Stokes 2008).

## Conclusion

The popularity of mobile telephony has brought with it new challenges in how to address public concerns about possible health impacts, but its benefits to society are enormous (Chapman et al. 1998). We conclude that in the absence of a scientifically plausible hazard from exposure to low-level RF, application of the PP is not appropriate to policy on the use of mobile phones and the siting of base stations. Indeed, we argue strongly that the conservative RF exposure standards, the technical features that minimize unnecessary exposures, support for ongoing research, regular review of safety recommendations, and availability of consumer information make mobile communications inherently precautionary.

In addition, governments and industry can do many things to better address public concern about the radio signals used for mobile communications. These include adoption of transparent and science-based regulations, continuing to support high-quality research, improving access to consumer relevant information, and ensuring that base station rollouts are conducted through the medium of an effec-tive communication program. Governments should educate the public about the scientific process with particular emphasis on the fact that not every publication, whether or not peer reviewed, is of equal scientific value. Government-appointed scientific review bodies should explain how study quality is assessed and the weights given to replication, dose response, and different study types in arriving at conclusions based on the entire body of relevant science (Scientific Committee on Emerging and Newly Identified Health Risks 2009; Vecchia 2007).

None of this is without difficulty, as shown by the mismatch between the intention of policy makers in making precautionary recommendations and the understanding of the public. This debate will continue to evolve over time, and challenges will remain, but society should not shrink from moving forward in a responsible manner with new and beneficial forms of wireless technology.

## Appendix 1: Working Definition of the Precautionary Principle

The following definition of the precautionary principle is taken from COMEST (2005; italics in the original):

When human activities may lead to morally unacceptable harm that is scientifically plausible but uncertain, actions shall be taken to avoid or diminish that harm.

*Morally unacceptable harm* refers to harm to humans or the environment that is

- threatening to human life or health, or
- serious and effectively irreversible, or
- inequitable to present or future generations, or
- imposed without adequate consideration of the human rights of those affected.

The judgment of *plausibility* should be grounded in scientific analysis. Analysis should be ongoing so that chosen actions are subject to review.

*Uncertainty* may apply to, but need not be limited to, causality or the bounds of the possible harm.

*Actions* are interventions that are undertaken before harm occurs that seek to avoid or diminish the harm. Actions should be chosen that are proportional to the seriousness of the potential harm, with consideration of their positive and negative consequences, and with an assessment of the moral implications of both action and inaction. The choice of action should be the result of a participatory process.
